# Synergistic Recruitment of Symbiotic Fungi by Potting and *Scleroderma bovista* Inoculation Suppresses Pathogens in Hazel Rhizosphere Microbiomes

**DOI:** 10.3390/microorganisms13051063

**Published:** 2025-05-02

**Authors:** Cheng Peng, Yuqing Li, Hengshu Yu, Hongli He, Yunqing Cheng, Siyu Sun, Jianfeng Liu

**Affiliations:** Jilin Provincial Key Laboratory of Plant Resource Science and Green Production, Jilin Normal University, Siping 136000, China; chengpeng000123@126.com (C.P.); 17833008176@163.com (Y.L.); 13500976936@163.com (H.Y.); honglihe2002@126.com (H.H.); chengyunqing1977@163.com (Y.C.); 15543327510@163.com (S.S.)

**Keywords:** hazel, microbial community, root exudates, *S. bovista*

## Abstract

This study explored how potted treatments (with and without *Scleroderma bovista* inoculation) shape rhizosphere microbial diversity in hazel across five soils using split-root cultivation. Three treatments (control, split-root, split-root with *S. bovista*) were analyzed for root growth and microbial dynamics. *S. bovista* inoculation consistently enhanced root parameters (number, tips) in all soils. Potted treatments (with and without *S. bovista* inoculation) altered microbial features (OTU/ASV), with only 0.9–3.3% of features remaining unchanged. At the class level, potting increased *Agaricomycetes* abundance while reducing *Sordariomycetes*, a trend amplified by *S. bovista*. Potting decreased species richness estimates (ACE and Chao1), while both treatments lowered diversity index (Shannon index). Potted treatments without *S. bovista* inoculation drove stronger shifts in species composition than inoculation. Findings reveal potting and *S. bovista* synergistically recruit symbiotic fungi via root exudates, establishing disease-suppressive communities that selectively inhibit pathotrophic fungi (particularly plant pathogen *Coniothyrium* and fungal parasite *Cladobotryum*) while roughly maintaining non-pathogenic saprotrophic microbes essential for organic matter decomposition. This work provides insights for optimizing hazel orchard management and ectomycorrhizal agent development.

## 1. Introduction

Mycorrhiza is a highly evolved mutualistic symbiosis between soil fungi and plant roots. In this mutually beneficial relationship, the host plant absorbs mineral nutrients from the soil through the symbiotic fungal hyphae, while the fungi obtain photosynthetic carbon assimilates from the host plant [1,2]. It is estimated that approximately 5 billion tons of photosynthetic products are fixed in soil by mycorrhizal fungi globally each year, playing a crucial role in the carbon and nitrogen balance of ecosystems [3,4]. Currently, it is widely recognized that mycorrhizal symbiosis enhances plant absorption and accumulation of soil mineral nutrients and promotes plant resistance to drought, flooding, salinity, diseases, and heavy metal stress in various ecosystems.

Hazel (*Corylus* spp.), a genus of plants in the Betulaceae family, is of significant economic importance. The hazelnut industry plays a vital role in supporting the economic development of mountainous regions in Northeast China, particularly in hazelnut-producing areas [5,6]. Studies have shown that *Corylus avellana* can form symbiotic relationships with arbuscular mycorrhizal fungi such as *Glomus intraradices*, as well as ectomycorrhizal fungi such as *Tuber melanosporum* and *Tuber albidum* [7,8]. These commercially available mycorrhizal fungi have been demonstrated to enhance root development, vegetative growth, nut yield, and oil production in *Corylus avellana*. However, suitable mycorrhizal preparations are currently lacking in the cultivation and production of hazel in China, which hinders the healthy development of the hazel industry.

There have been several studies on the microbial diversity of hazelnut rhizosphere soil. Some studies have compared the diversity of hazel rhizosphere soil across different seasons and identified available potassium as a key factor influencing fungal diversity in hazel rhizosphere soil [9,10]. Another study investigated the effects of orchard grass on fungal diversity in hazel orchard soils and suggested that living cover can increase the relative abundance of symbiotic trophic microorganisms in hazelnut orchards [11]. To date, it has been widely recognized that hazel can form ectomycorrhizal associations. Several studies have explored the inoculation of ectomycorrhizal fungi in hazel orchards to promote plant growth [7,12,13,14]. However, these ectomycorrhizal fungi do not appear to have been isolated directly from hazel roots.

In recent years, significant progress has been made in the study of an ectomycorrhizal fungus associated with hazel [5,6,7,8,9,10,11]. A naturally occurring ectomycorrhizal fungus was discovered in the hazel production areas of Northeast China. Through isolation, purification, sequencing, and sequence alignment, this fungus was identified as *Scleroderma bovista*. Outdoor pot experiments with hazel seedlings inoculated with *S. bovista* demonstrated that the fungus strongly promotes the growth of both above-ground and underground parts of the seedlings [3,4]. These findings suggest that *S. bovista* has the potential to be developed into a specialized mycorrhizal preparation for hazel cultivation. However, the hazelnut industry in Northeast China currently faces specific challenges in adopting mycorrhizal preparations, as none have been effectively applied in local production and cultivation practices to date. Microbial diversity varies significantly across soils with different physicochemical properties. However, the impact of root exudates on rhizosphere microbial diversity in hazel cultivation, particularly in soils with varying microbial profiles, remains poorly understood. Furthermore, it has yet to be experimentally verified whether hazel roots can establish mycorrhizal associations with *S. bovista* and how the diversity of rhizosphere soil microbes responds to *S. bovista* inoculation in soils with distinct microbial compositions.

Plant rhizosphere fungi include both harmful fungi that inhibit plant growth and beneficial fungi that promote it. Harmful fungi are often associated with yield reduction during continuous cropping. They primarily secrete plant toxins, compete for nutrients, and may also inhibit mycorrhizal function, thereby suppressing plant growth [15]. In contrast, the beneficial microbial community in the rhizosphere includes biocontrol fungi, fungi capable of producing plant growth hormones, and nitrogen-fixing bacteria [15]. In agricultural ecosystems, fully harnessing the biological potential of these fungi can help reduce fertilizer and pesticide inputs, promote plant growth, minimize environmental pollution, and support sustainable agricultural development [16]. In the hazel-producing areas of Northeast China, significant differences in microbial diversity have been observed among different hazel orchards. However, it remains unclear whether inoculation with *S. bovista* can inhibit harmful fungi and promote the proliferation of beneficial fungi, thereby enhancing hazel growth. Understanding the impact of *S. bovista* on the microbial diversity of hazel rhizosphere soil is crucial to determine its potential as a specialized ectomycorrhizal preparation for hazel cultivation. Detailed and in-depth research is needed to address these questions.

We hypothesize that both hazel root exudates and *S. bovista* inoculation alter the diversity and composition of rhizosphere microbial communities, enhance the relative abundance of symbiotic fungi, and promote root growth in hazel seedlings. In this study, five soil samples from hazelnut gardens in geographically distinct locations were selected as cultivation substrates. Subsequent microbial community analysis confirmed significant diversity gradients among these soils. We evaluated the effects of *S. bovista* on root growth parameters and compared the microbial diversity of the original soil, rhizosphere soil, and rhizosphere soil following *S. bovista* inoculation. The aim of this study was to investigate the relationship between root exudates and *S. bovista* inoculation on the diversity of rhizosphere fungi in hazel, providing a scientific foundation for hazelnut orchard management and the development of ectomycorrhizal agents.

## 2. Materials and Methods

### 2.1. Experimental Materials and Design

**Figure 1:**
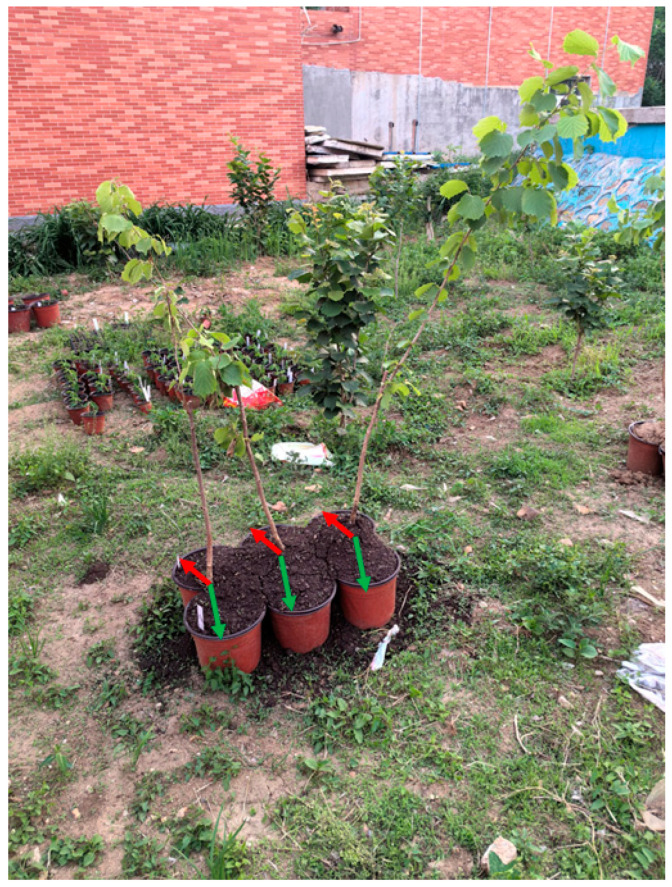
Split-root cultivation system of hazelnut seedlings. *Note*: Red and green arrows indicate the distribution of a single seedling’s root system between two pots.

The strain of *Scleroderma bovista* was patent-deposited at the China General Microbiological Culture Collection Center (CGMCC) under the accession number CGMCC 40054. Genome sequencing and assembly of *S. bovista* have been completed, and the genomic sequence has been submitted to the National Center for Biotechnology Information (NCBI) public database with the accession number PRJNA1048296. The outdoors split-root cultivation experiment was conducted from May to September 2024 at Jilin Normal University (43°9′ N, 124°20′ E). Five different soil types were collected from five hazel orchards in Jilin Province. The soil was collected from the inter-row area between two rows of hazel trees (topsoil layer, 0–20 cm), avoiding the rhizosphere zone. This sampling strategy was designed to isolate bulk soil microbial communities unaffected by root exudation, as the study specifically aimed to investigate the targeted effects of hazel root-derived substances under controlled experimental conditions. The basic physical and chemical properties of the soil are listed in Table S1. The collected soil samples are divided into three parts, one for detecting the physiochemical properties of the soil, one for sequencing the microbial diversity of the soil, and one for split-root cultivation experiments. The hybrid hazel (*C. heterophylla* × *C. avellana*) variety ‘Dawei’, a widely cultivated commercial variety in the hazel production areas of Northeast China, was used for the experiment. Two-year-old ‘Dawei’ seedlings were selected for the pot experiments. The five soil types were labeled A, B, C, D, and E. Three treatments were established for the experiment: (1) Blank control (denoted as 0): After collection, the soil was not used for potted hazel seedlings. (2) Split-root cultivation and potted treatment without inoculation (denoted as 1). (3) Split-root cultivation and potted treatment with *S. bovista* inoculation (denoted as 2). Hazel seedlings were carefully transplanted to the central junction, with roots manually arranged into both compartments. The substrate was regularly moistened to 70% field capacity using tap water at 5-day intervals. The growth of *S. bovista* mycelium and its symbiotic relationship with the root system were inspected at 20-day intervals using the trench-profile method. The cultivation setup for hazel seedlings is illustrated in Figure 1. The plastic pots used in the experiment had a capacity of approximately 4.4 L, with upper and lower bottom diameters of 12.7 cm and 9.5 cm, respectively, and a height of 11.4 cm. The vegetative mycelium of *S. bovista* was cultured and propagated in MMN medium (Modified Melin-Norkrans Medium). Ten cubic blocks containing actively growing mycelium were inoculated adjacent to the root system at a depth of 5.0 cm on 5 May 2024. The inoculation method and quantity were based on the protocol described in the literature [6]. Each treatment included three biological replicates and each replicate included three hazel seedlings.

### 2.2. Measurement and Analysis of Root Parameters of Hazel Seedlings

In the split-root cultivation experiment, the root system of each hazel seedling was subjected to two treatments: *S. bovista* inoculation and non-inoculation. This study utilized five soil types, resulting in a total of 10 treatments. Each treatment included three biological replicates, yielding a total of 30 root samples. Approximately 2.0 g of the hazel root system was cut from the top using scissors, and root system images were captured using WinRhizo (Ver. 5.0A; Regent Instruments Inc., Quebec, QC, Canada) to measure and calculate root-related indicators on September 10th, including root number, root tip number, total length, root diameter, surface area, and number of forks. Statistical analyses were performed using the ANOVA procedure in SAS version 8.01 (SAS Institute, Inc., Cary, NC, USA). The means of root-related indicators for different treatments were compared using the least significant difference (LSD) *t*-test at a 5% significance level.

### 2.3. Extraction and Sequencing of Soil DNA

Rhizosphere soil from hazel seedlings was collected on September 10th, with 5.0 g of soil taken for each replicate. The collected soil samples were transferred to centrifuge tubes and then placed in liquid nitrogen for 1 h. Subsequently, the samples were transported on dry ice to Beijing Biomarker Technologies Co., Ltd. for soil microbial diversity analysis. DNA extraction from the soil samples was performed using the E.Z.N.A. Soil DNA Kit (OMEGA, Norcross, GA, USA) according to the manufacturer’s instructions. After extracting the total DNA from the samples, ITS1 primers (F: CTTGGTCATTTAGAGAAGTAA; R: GCTGCGTTTTGATGATGC) were designed based on the fungal conserved region using software Primer Premier 5.0. Sequencing adapters were added to the ends of the primers, and PCR amplification was conducted with the following thermal parameters: initial DNA denaturation at 95 °C (1 min), succeeded by 25 amplification cycles consisting of denaturation (95 °C, 20 s), primer annealing (56 °C, 30 s), and elongation (65 °C, 2 min), culminating in a terminal extension at 65 °C for 5 min. Following PCR amplification, the amplicons underwent systematic library preparation: First, purification was performed using AMPure XP magnetic beads (Beckman Coulter, Brea, CA, USA; 1.0 × bead-to-sample ratio) to eliminate primer dimers and fragments < 100 bp [17]. Subsequently, DNA concentrations were quantified via Qubit 4.0 Fluorometer (Thermo Fisher Scientific, Waltham, MA, USA) with the Qubit dsDNA HS Assay Kit, followed by normalization to 4 nM using 10 mM Tris-HCl (pH 8.5) for equimolar pooling. Library integrity was validated through an Agilent 2100 Bioanalyzer (Agilent Technologies, Santa Clara, CA, USA) equipped with high-sensitivity DNA chips [18]. Qualified libraries were then subjected to paired-end sequencing (2 × 150 bp) on the Illumina NovaSeq 6000 platform following standard manufacturer protocols.

Data preprocessing primarily consisted of the following two steps: (1) First, the raw reads obtained from sequencing were filtered using the software Trimomatic (v0.33) [19]. Subsequently, primer sequences were identified and removed using the software Cutadapt (v1.9.1) [20], resulting in clean reads free of primer sequences. (2) The DADA2 method [21] within QIIME (v2020.6.0) [22] was employed for denoising, concatenation of paired-end sequences, and removal of chimeric sequences to obtain the final valid data (non-chimeric reads). DADA2-generated amplicon sequence variants (ASVs) enhance taxonomic resolution by 1–2 orders of magnitude compared to traditional OTU clustering methods [21,22,23].

### 2.4. Sequencing Data Analysis

ASVs (amplicon sequence variants) represent exact biological sequences and resolve differences at the single-nucleotide level, enabling relatively precise identification of species or even strains within samples. The DADA2 algorithm [21] in the QIIME (v2020.6.0) software [21] was used to denoise sequences and generate ASVs. Taxonomic annotation of the feature sequences (OTUs/ASVs) was performed using the UNITE (v8.0) [24] species annotation database (https://unite.ut.ee/) as a reference on 10 January 2025, combining Bayesian inference with the BLAST (Version 2.9.0) alignment method. This process provided species classification information corresponding to each feature and enabled the calculation of community composition for each sample at various taxonomic levels (phylum, class, order, family, genus, species). The QIIME (v2020.6.0) software [22] was used to generate species abundance tables at different classification levels, and Excel and R language tools were employed to create bar charts and Venn diagrams to visualize the statistical results of species composition and relative abundance. Alpha and Beta diversity indices were evaluated using QIIME (v2020.6.0) [22]. Specifically, alpha diversity metrics included the Abundance-based Coverage Estimator (ACE) [25], Chao1 richness estimator [26], Simpson diversity index [27], and Shannon–Wiener diversity index [28]. For Beta diversity analysis, the binary Jaccard algorithm was applied to calculate the distance between samples and derive beta values. Hierarchical clustering of samples was performed using the unweighted pair group method with arithmetic mean (UPGMA) in the R language tool to assess similarities in species composition among samples [29].

FUNGuild (Fungi Functional Guild) is an annotation tool used in parsing fungal communities in ecological studies. It utilizes a simple and consistent method to classify large sequence datasets into categories with ecological meaning. Based on trophic mode, fungal can be classified into pathotroph, symbiotroph and saprotroph. In the present study, microbial diversity data from all 45 soil samples were divided into three groups: Group 0, Group 1, and Group 2, each containing 15 samples. Group 0 consisted of 15 samples from treatments A0, B0, C0, D0, and E0. Group 1 included 15 samples from treatments A1, B1, C1, D1, and E1, and Group 2 comprised 15 samples from treatments A2, B2, C2, D2, and E2. Fungal phenotype prediction was conducted using BMKCloud on 20 December 2024 (www.biocloud.net). Through pairwise comparisons between Group 0 vs. Group 1 and Group 1 vs. Group 2, the effects of root exudates and *S. bovista* inoculation on the relative abundance of pathotroph, symbiotorph, and saprotroph microorganisms were evaluated.

### 2.5. Statistical Analysis

The experimental data were analyzed for variance using the ANOVA procedures of SAS 8.01, and significance was tested with Duncan’s new complex extreme difference method. If the data were expressed as percentages, an arcsine square root transformation was applied prior to analysis of variance.

## 3. Results

### 3.1. Effects of S. bovista Inoculation on Root Index in Split-Root Cultivation Experiment

**Figure 2:**
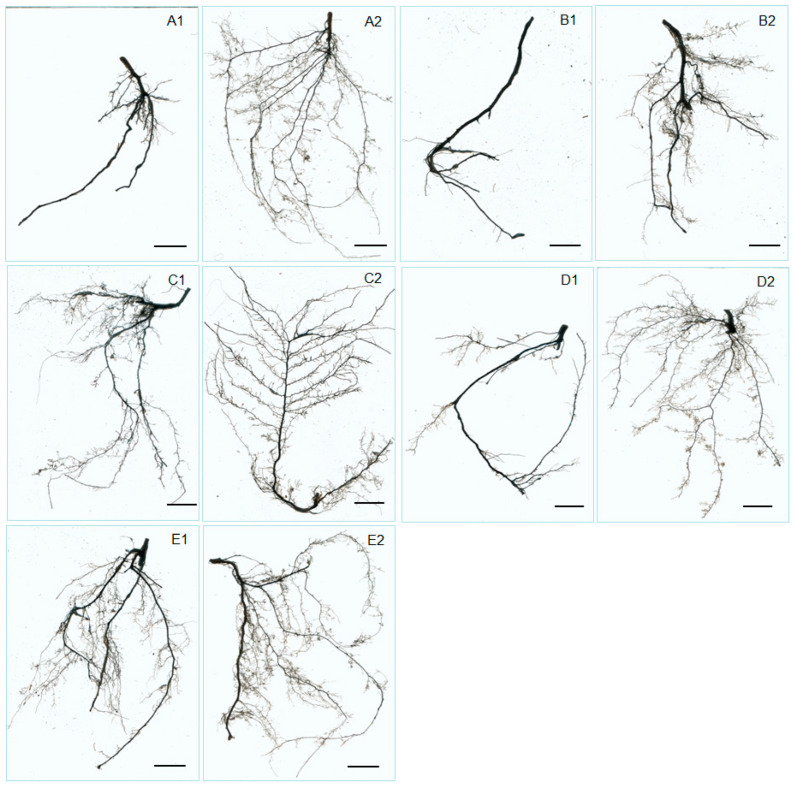
Effects of *S. bovista* inoculation on root morphology of hazel seedlings. (**A1**–**E2**) Five soil types. “1”: Control (no inoculation); “2”: *S. bovista* inoculation. Roots (2.0 g fresh weight) were imaged using WinRhizo. Scale bar = 3.0 cm.

Split-root cultivation of hazel seedlings was conducted to evaluate the effects of *S. bovista* inoculation on root growth. In the paired comparison of A1 (control, without *S. bovista* inoculation) and A2 (with *S. bovista* inoculation), treatment A2 exhibited a higher number of root branches, increased root density, and a substantially larger root absorption area (Figure 2(A1,A2)). Similar trends were observed in the pairwise comparisons of B1 vs. B2 (Figure 2(B1,B2)), C1 vs. C2 (Figure 2(C1,C2)), D1 vs. D2 (Figure 2(D1,D2)), and E1 vs. E2 (Figure 2(E1,E2)). These results demonstrate that *S. bovista* inoculation consistently enhanced root development across all five soil types, as evidenced by increased root branching and absorption area.

**Figure 3:**
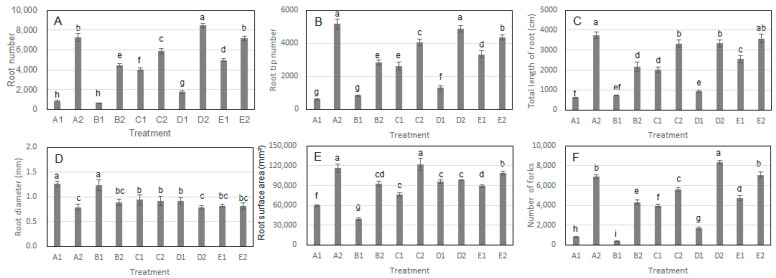
Effects of *S. bovista* inoculation on root architecture indices in split-root systems. (**A**) Root number; (**B**) Root tip density; (**C**) Total root length; (**D**) Root diameter; (**E**) Root surface area; (**F**) Root forks. *Note*: Letters A–E denote five soil types. “1”: Control; “2”: *S. bovista* inoculation. Lowercase letters indicate significant differences (*p* ≤ 0.05; ANOVA).

In the comparison of A1 and A2, treatment A2 showed significant increases in root number, root tip number, total root length, root surface area, and the number of root forks (Figure 3A–C,E,F). Additionally, the root diameter in A2 was significantly smaller than that in A1 (Figure 2(A1,A2)). Similar patterns were observed in the comparisons of B1 vs. B2, C1 vs. C2, D1 vs. D2, and E1 vs. E2 (Figure 3D). Specifically, *S. bovista* inoculation significantly increased root number, root tip number, total root length, root surface area, and the number of root forks, but had no significant effect on root diameter. Collectively, these findings indicate that *S. bovista* inoculation significantly promotes root growth parameters, including root number, root tip number, total root length, root surface area, and the number of forks, across all five soil types.

### 3.2. Raw Data Quality Control and OTU/ASV Analysis

A total of 45 soil samples were sequenced, generating 3,054,213 raw reads. The raw data for the hazelnut rhizosphere microbial genome have been deposited in the NCBI SRA database under accession number PRJNA1224535. Primer sequences were identified and removed, resulting in 2,735,611 clean reads. After denoising, concatenation of double-ended sequences, and removal of chimeric sequences, 2,616,082 high-quality reads (non-chimeric reads) were retained. On average, each soil sample yielded 58,135 non-chimeric reads (Table S2). The total number of features (OTUs/ASVs) and feature reads were 9528 and 2,613,496, respectively (Table S3). Per soil sample, the average number of features (OTUs/ASVs) and feature reads were 212 and 58,078, respectively.

### 3.3. Impact of Potting and S. bovista Inoculation on Soil Feature Number

**Figure 4:**
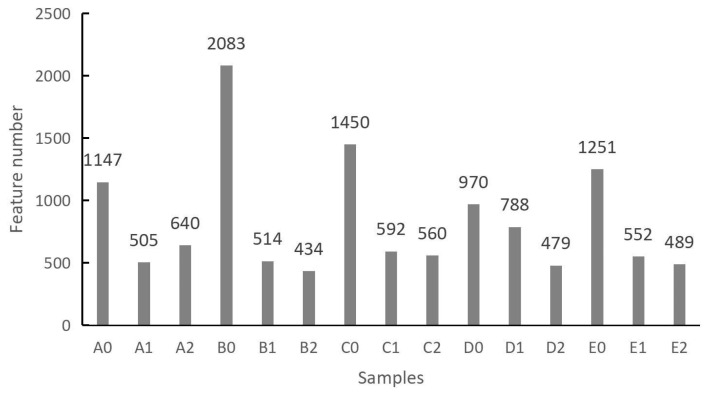
Statistics of feature numbers across treatments. *Note*: A–E: Soil types. “0”: Unplanted control; “1”: Planted without inoculation; “2”: Planted with *S. bovista* inoculation. Feature: Operational taxonomic unit (OTU)/amplicon sequence variant (ASV).

Substantial differences in feature numbers were observed among the five soil samples, ranging from 1147 to 2083 (Figure 4; Table S4). After four months of potting, the retrieved soil exhibited a notable reduction in feature numbers, with decreases ranging from 18% to 75%. Specifically, compared to D0, the feature number in D1 decreased by 18%, while the feature number in B1 decreased by 75% relative to B0 (Figure 4). In the split-root cultivation experiment, *S. bovista* inoculation also significantly influenced soil feature numbers. Compared to B1, C1, D1, and E1, the feature numbers in B2, C2, D2, and E2 decreased by 5% to 39%. In contrast, the feature number in A2 increased by 26% relative to A1 (Figure 4).

**Figure 5:**
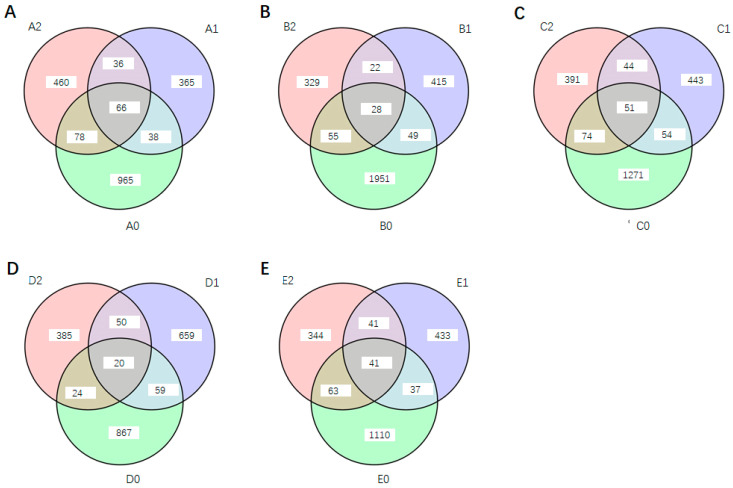
Venn diagram of microbial features across different treatments. (**A**) Treatments of A0, A1 and A2; (**B**) Treatments of B0, B1 and B2; (**C**) Treatments of C0, C1 and C2; (**D**) Treatments of D0, D1 and D2; (**E**) Treatments of E0, E1 and E2. *Note*: A–E: Soil types. “0”: Unplanted control; “1”: Planted without inoculation; “2”: Planted with *S. bovista* inoculation. The numbers in the Venn diagram represent the identified number of features (OTU/ASV).

A0 and A1 represent soil samples without and with potting, respectively. The quotient of the intersection and union of their feature numbers was calculated to be 6.7%. Similarly, the corresponding quotients for soils B, C, D, and E were 3.1%, 5.4%, 4.7%, and 4.5%, respectively (Figure 5). These calculations indicate that potting significantly alters soil feature numbers, with only 3.1% to 6.7% of features remaining unchanged before and after potting.

Both A1 and A2 were potted treatments, with A1 serving as the non-inoculated control and A2 receiving *S. bovista* inoculation. The quotient of the intersection and union of their feature numbers was 9.7%. The corresponding values for soils B, C, D, and E were 5.6%, 9.0%, 5.8%, and 8.6%, respectively (Figure 5). These results suggest that *S. bovista* inoculation significantly impacts soil feature numbers, with only 5.6% to 9.7% of features remaining unchanged after inoculation.

Finally, the intersection and union of feature numbers across the three treatments (A0, A1, and A2) yielded a quotient of 3.3%. The corresponding values for soils B, C, D, and E were 0.9%, 2.1%, 1.0%, and 2.0%, respectively (Figure 5). These findings demonstrate that both potting and *S. bovista* inoculation have a substantial impact on soil feature numbers, with only 0.9% to 3.3% of features remaining consistent across all treatments.

### 3.4. Impact of Hazel Seedling Potting on the Relative Abundance of Soil Fungi

**Figure 6:**
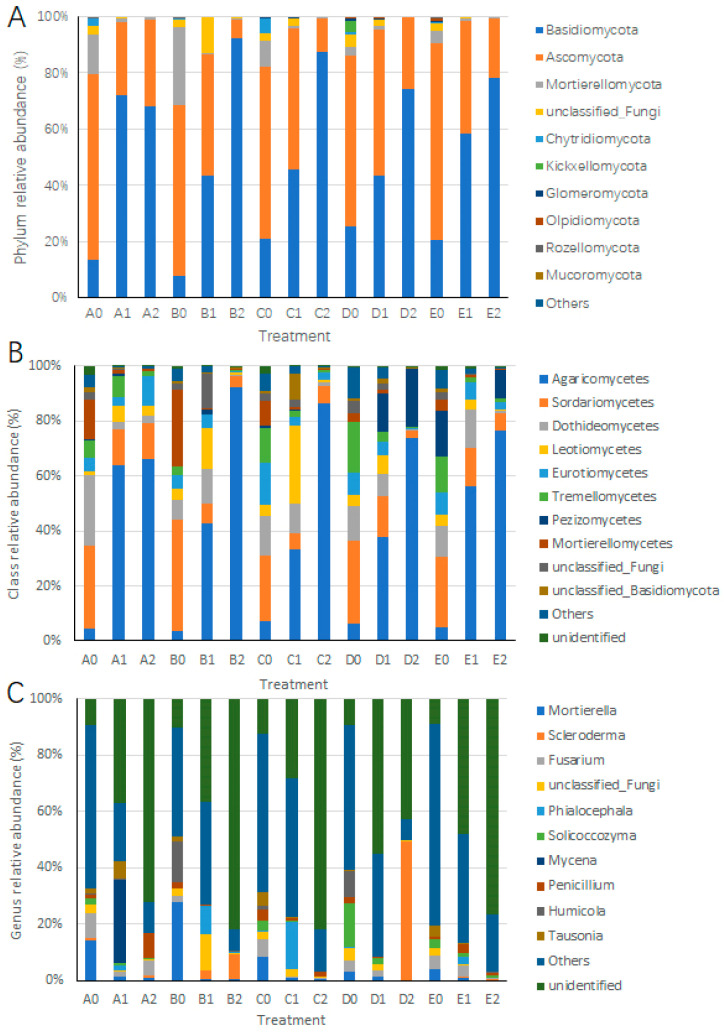
Taxonomic shifts in rhizosphere microbiota at phylum (**A**), class (**B**), and genus (**C**) levels. *Note*: *X*-axis: treatments; *Y*-axis: relative abundance (%). Colors denote taxa (top 10 per rank). A–E: Soil types. “0”: Unplanted control; “1”: Planted without inoculation; “2”: Planted with *S. bovista* inoculation.

At the phylum level, we evaluated the impact of hazel seedling potting on the relative abundance of soil fungi (Table S5). Compared to A0, the relative abundance of Basidiomycota in A1 increased significantly by 58.75%, while the relative abundances of Ascomycota and Mortierellomycota decreased by 40.32% and 12.84%, respectively. Similar patterns were observed in the other four soil types. Compared to B0, C0, D0, and E0, the relative abundance of Ascomycota in B1, C1, D1, and E1 decreased by 8.84% to 29.74%, while the relative abundance of Mortierellomycota decreased by 1.63% to 27.42% (Figure 6A). Overall, the potting of hazel seedlings led to an increase in the relative abundance of Basidiomycota and a decrease in the relative abundances of Ascomycota and Mortierellomycota across all five soil types.

**Figure 7:**
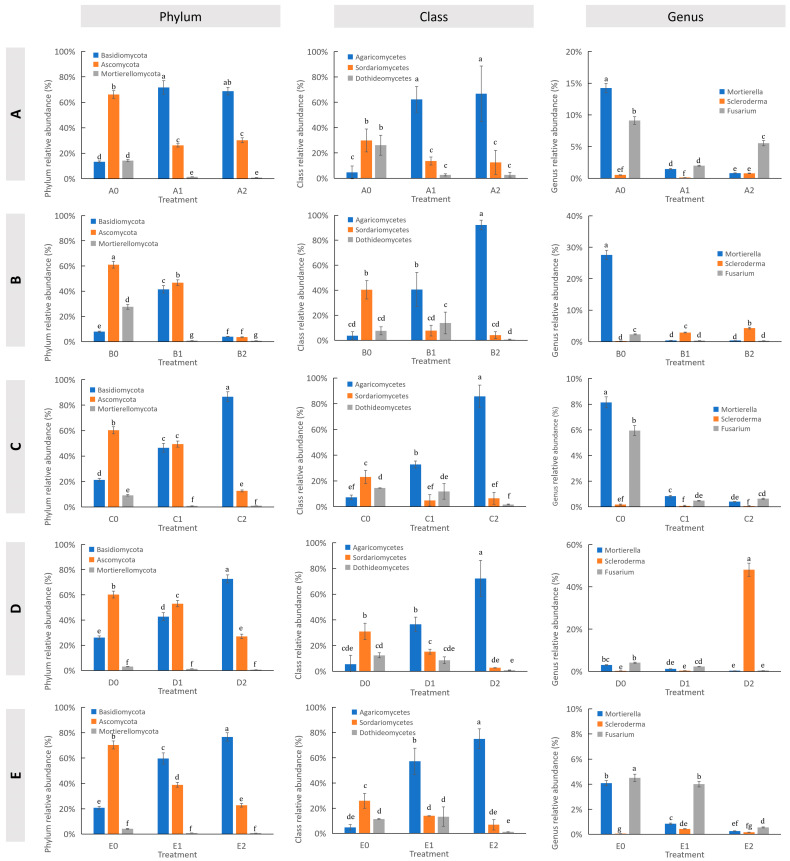
Dominant taxonomic groups (top 3) at phylum, class, and genus levels. (**A**) Soil type A; (**B**) Soil type B; (**C**) Soil type C; (**D**) Soil type D; (**E**) Soil type E. *Note*: *X*-axis: treatments; *Y*-axis: relative abundance (%). Lowercase letters indicate significant differences (*p* ≤ 0.05). A–E: Soil types. “0”: Unplanted control; “1”: Planted without inoculation; “2”: Planted with *S. bovista* inoculation.

At the class level, the potting of hazel seedlings increased the relative abundance of *Agaricomycetes* in all five soil types, with increases ranging from 26.31% to 59.02%, while the relative abundance of *Sordariomycetes* decreased by 11.6% to 33.19% (Figure 6B and Figure 7; Table S4). Significant differences in the relative abundances of *Agaricomycetes* and *Sordariomycetes* were observed in the pairwise comparisons of A1 vs. A0, B1 vs. B0, C1 vs. C0, D1 vs. D0, and E1 vs. E0 (Figure 7).

At the genus level, the potting of hazel seedlings significantly decreased the relative abundance of *Mortierella* (61–98%) and *Fusarium* (10–93%) in all five soil types (Figure 6C and Figure 7). The potting of hazel seedlings significantly increased that of *Scleroderma* in soil types B and D (Figure 6C and Figure 7).

### 3.5. Impact of S. bovista Inoculation on the Relative Abundance of Soil Fungi

At the phylum level, *S. bovista* inoculation increased the relative abundance of Basidiomycota by 19.62% to 48.82% and decreased the relative abundance of Ascomycota by 18.67% to 38.11% in soils B, C, D, and E (Figure 6A).

At the class level, *S. bovista* inoculation increased the relative abundance of *Agaricomycetes* by 20.60% to 52.98% in soils B, C, D, and E (Figure 6B). Significant differences in the relative abundance of *Agaricomycetes* were observed in the pairwise comparisons of B2 vs. B1, C2 vs. C1, D2 vs. D1, and E2 vs. E1 (Figure 7; Table S4).

At the genus level, *S. bovista* inoculation significantly decreased the relative abundance of *Mortierella* (44–93%) in soil types A, C, D, and E, while increasing that of *Scleroderma* in soil types A, B, and D (Figure 6C and Figure 7).

### 3.6. Impact of Hazel Seedling Potting and S. bovista Inoculation on Alpha Diversity of Soil Samples

**Figure 8:**
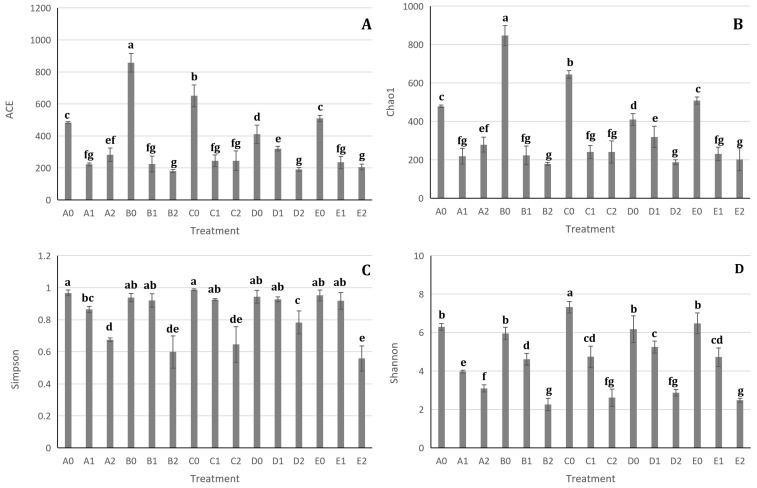
Impact of hazel seedling potting and *S. bovista* inoculation on alpha diversity across 15 treatments. (**A**) ACE; (**B**) Chao1; (**C**) Simpson; (**D**) Shannon. *Note*: ACE: Abundance-based coverage estimator. A–E: Soil types. “0”: Unplanted control; “1”: Planted without inoculation; “2”: Planted with *S. bovista* inoculation. Letters indicate significant differences (*p* = 0.05).

The effects of hazel seedling potting and *S. bovista* inoculation on alpha diversity are shown in Figure 8. For soil samples A1 and A2, both ACE and Chao1 indices were significantly lower than those of A0, though no significant difference was observed between A1 and A2 (Figure 8A,B). Similar trends were observed in the other four soil types (Figure 8A,B). These results indicate that hazel seedling potting significantly reduced alpha diversity indices (ACE and Chao1), which measure species richness (i.e., the total number of microbial species in the community), while *S. bovista* inoculation had no significant impact on these metrics. This suggests that potting reduced microbial species richness in rhizosphere soil, whereas fungal inoculation did not alter species richness.

In pairwise comparisons of A1 vs. A0, B1 vs. B0, C1 vs. C0, D1 vs. D0, and E1 vs. E0, the latter treatments exhibited lower Shannon and Simpson indices than the former (Figure 8C,D). Similarly, in comparisons of A2 vs. A1, B2 vs. B1, C2 vs. C1, D2 vs. D1, and E2 vs. E1, the Shannon and Simpson indices of inoculated treatments (A2–E2) were significantly lower than those of non-inoculated controls (A1–E1) (Figure 8C,D). Since higher Shannon and Simpson index values indicate greater species diversity, these results demonstrate that both hazel seedling potting and *S. bovista* inoculation reduced species diversity in rhizosphere soil to varying degrees.

### 3.7. Impact of Hazel Seedling Potting and S. bovista Inoculation on Beta Diversity of Soil Samples

**Figure 9:**
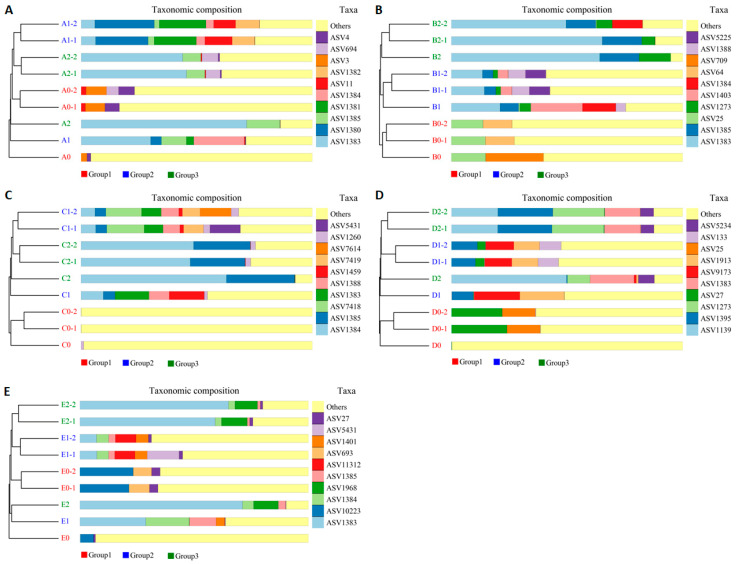
Beta diversity analysis using UPGMA clustering (**A**–**E**) for soil groups A0–A2 to E0–E2. *Note*: Cluster trees paired with abundance bar charts (top 10 taxa). Branch length reflects compositional similarity. A0: Triplicate controls; A1/A2: Triplicate treatments. A–E: Soil types. “0”: Unplanted control; “1”: Planted without inoculation; “2”: Planted with *S. bovista* inoculation.

Beta diversity analysis (UPGMA clustering tree and abundance bar chart; Figure 9) revealed differences in species composition similarity among the 45 soil samples. Shorter branch lengths between samples indicate greater similarity in species composition. In the A0–A1–A2 comparison, A1 and A2 clustered closely with short branch lengths, whereas A0 diverged significantly from both (Figure 9A). Similar clustering patterns were observed in soils B–E (Figure 9B–E). These findings suggest that hazel seedling potting substantially altered rhizosphere soil species composition, while *S. bovista* inoculation had minimal additional impact.

### 3.8. Fungal Phenotype Prediction

**Figure 10:**
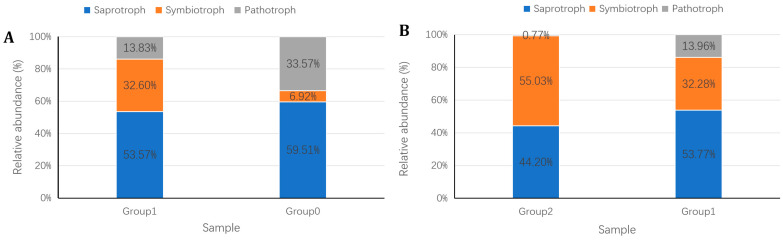
FUNGuild-based functional profiling of fungal communities. (**A**) Potting effects; (**B**) *S. bovista* inoculation effects. *Note*: *X*-axis: Treatment groups (0: control; 1: non-inoculated; 2: inoculated); *Y*-axis: Relative abundance (%). Guild classification: Pathotroph, symbiotroph, saprotroph. Group 0 (A0–E0), Group 1 (A1–E1), Group 2 (A2–E2); n = 15 per group.

Compared to Group 0 (control), Group 1 (potted soil) exhibited a slight decrease in saprotroph abundance (5.94% reduction), a substantial decline in pathotroph abundance (19.74% reduction), and a marked increase in symbiotroph abundance (25.68% increase, Figure 10A; i.e., *Phialocephala*, *Cadophora*, *Scleroderma*, and *Astraeus*, Table S6). These shifts indicate that hazel root exudates promote symbiotic fungi while suppressing saprotrophic (i.e., *Thelonectria*, *Penicillium simplicissimum*; Table S6) and pathogenic fungi (i.e., *Coniothyrium* and *Cladobotryum*; Table S6). In Group 2 (*S. bovista*-inoculated soil), symbiotroph abundance increased by 22.74% compared to Group 1 (i.e., *Scleroderma*, *Phialocephala*, *Cadophora*, *Rhizophagus* and Glomeraceae, Table S7), consistent with the observed enrichment of the ectomycorrhizal fungus *S. bovista*. Concurrently, saprotroph abundance decreased slightly (9.57% reduction) and pathotroph abundance declined significantly (13.19% reduction; Figure 10B). These results demonstrate that *S. bovista* inoculation further enhances symbiotic fungi proliferation while inhibiting saprotrophic and pathogenic fungi. Collectively, both hazel root exudates and *S. bovista* inoculation promote symbiotic fungal growth and suppress saprotrophic and pathogenic fungi in rhizosphere soil.

## 4. Discussion

A previous study [10] analyzed the seasonal variation in rhizosphere soil microbial diversity in hazelnut species (*C. heterophylla*, *C. kweichowensis*, *C. avellana*, and *C. heterophylla* × *C. avellana*) at a sampling site in Yanqing District, Beijing, China. In contrast, our experimental site was located at Jilin Normal University, approximately 900 km from the Yanqing site. Here, the effects of potted hazelnut seedlings and *S. bovista* inoculation on soil microbial diversity from different sources were investigated using *C. avellana* and *C. heterophylla × C. avellana* as materials. Surprisingly, despite the geographical distance and significant differences in climate and soil conditions between the two experiments, the dominant microbial communities in the hazelnut rhizosphere soil were identical at the class level, although their relative abundances varied significantly. The dominant microbial classes included *Agaricomycetes*, *Sordariomycetes*, *Dothideomycetes*, *Leotiomycetes*, *Eurotiomycetes*, *Tremellomycetes*, *Pezizomycetes*, and *Mortierellomycetes* (Figure 6B; [10]). *Agaricomycetes*, which include many mushroom species, showed an increase in relative abundance, suggesting more favorable soil conditions for mushroom growth. *Agaricomycetes* are widely involved in the decomposition of organic matter, such as wood, and often form mycorrhizal symbioses with plant roots, playing a crucial role in ecosystem nutrient cycling and energy flow [10]. Plants secrete or release various substances into the growth medium through different parts of their roots during their growth process. These substances include low-molecular-weight organic compounds, high-molecular-weight adhesive compounds, and root cell abscission products and their decomposition products, as well as gases, protons, and nutrient ions. Therefore, we propose that hazelnut root exudates have a decisive influence on the species composition of rhizosphere soil fungi.

This study revealed that root exudates from hazelnut (*Corylus* spp.) significantly reduced the relative abundance of *Mortierella* (61–98%) and *Fusarium* spp. (10–93%) in soil ecosystems. Notably, *Fusarium* species encompass multiple soil-borne pathogens (e.g., *Fusarium oxysporum*), with hazelnut root rot primarily attributed to *F. oxysporum* and *F. solani*, whose infection causes browning of root xylem and biomass loss [30,31,32]. Intriguingly, analogous to the rhizospheric proliferation and colonization dynamics of *Burkholderia* sp. enhanced by ginger-derived exudates in chrysanthemum (as demonstrated in previous studies) [33], our findings suggest that hazelnut may recruit antagonistic microbiota through root exudation to suppress *Fusarium* pathogens, thereby directly alleviating root disease pressure. Of particular ecological significance is the marked decrease in *Mortierella* populations, typically characterized as saprophytic fungi involved in organic matter decomposition and lipid metabolism [34,35]. This observed suppression implies that hazelnut root exudates may selectively inhibit specific saprotrophs, potentially optimizing rhizospheric carbon allocation strategies through microbial community restructuring.

Root exudates not only provide essential nutrition and protection for plants but also create a nutrient-rich environment for rhizosphere fungi. Symbiotic fungi form close relationships with plants, relying on root exudates as their primary carbon and energy sources [36]. The type and quantity of root exudates directly influence the community structure and abundance of symbiotic fungi. An increase in root exudate secretion attracts more symbiotic fungi, leading to their proliferation. Additionally, root exudates can indirectly promote microbial growth by improving the soil structure and environment [37]. In this study, the relative abundance of symbiotic trophic fungi in Group 1 increased by 25.68% compared to Group 0 (Figure 10; Table S6). These fungi obtain nutrients through resource exchange with host cells. Specifically, hazelnut root exudates significantly increased the relative abundance of endophytic fungi (*Phialocephala*, *Cadophora*) and ectomycorrhizal fungi (*Scleroderma*, and *Astraeus*) (Table S6). *Phialocephala*, an endophytic fungus commonly found in the roots of plants such as blueberries and larch, has been shown to promote plant growth and nutrient absorption [38,39,40]. It also plays a vital role in forest ecosystems by decomposing organic matter and facilitating the cycling of carbon, nitrogen, and phosphorus [40]. Similarly, *Cadophora*, a dominant genus in soil and plant root fungal communities, is closely associated with soil nutrient content and plant development [41]. The increased abundance of *Phialocephala* and *Cadophora* in the hazelnut rhizosphere likely enhances nutrient absorption and plant growth.

Ectomycorrhizal fungi, such as *Scleroderma*, are known to enhance plant resistance to pathogens, improve drought tolerance, and promote nutrient uptake [42]. *Scleroderma* forms mutualistic symbiotic relationships with plant roots, expanding root absorption areas and improving mineral nutrient uptake, thereby enhancing plant growth and stress tolerance [43,44]. The increased relative abundance of *Scleroderma* in the hazelnut rhizosphere aligns with the effects of *S. bovista* inoculation observed in this study, confirming that *S. bovista* promotes hazelnut root development. Additionally, hazelnut root exudates increased the relative abundance of arbuscular mycorrhizal fungi, such as *Rhizophagus* and Glomeraceae. These fungi form symbiotic relationships with plant roots, enhancing nutrient absorption, particularly phosphorus, and improving plant stress resistance [43,44]. They also contribute to soil structure improvement and nutrient retention [43]. The increased abundance of Glomeraceae fungi, particularly the genus *Rhizophagus*, suggests their potential role in promoting hazelnut growth and nutrient absorption, which could lead to the development of hazelnut-specific fungal agents.

In this study, hazelnut root exudates led to a slight decrease (5.94%) in the relative abundance of saprotrophs, including leaf saprotroph *Thelonectria* and wood saprotroph *Penicillium simplicissimum* (Figure 10; Table S6), which are involved in leaf and wood degradation [45,46]. Additionally, a substantial decrease (19.74%) was observed in the relative abundance of pathotrophic fungi, including plant pathogen *Coniothyrium* and fungal parasite *Cladobotryum*. *Coniothyrium* plays a dual role in ecosystems: As a plant pathogen, it threatens agricultural production, while as a wood saprotroph, it contributes to organic matter decomposition and nutrient cycling [43]. *Cladobotryum* primarily infects and destroys edible fungi but may also participate in ecosystem nutrient cycling [47]. The specific mechanisms by which root exudates reduce the abundance of saprotrophic and pathotrophic fungi remain unclear. Some studies suggest that plants recruit beneficial microbes through root exudates under stress conditions, indirectly affecting the abundance of saprotrophic and pathotrophic fungi [48,49]. Hazelnuts produce secondary metabolites such as paclitaxel, known for its anti-tumor properties [50], which may also inhibit microbial proliferation [51]. However, further research is needed to identify the specific components in root exudates responsible for these effects.

*S. bovista* inoculation promoted the reproduction and enrichment of symbiotic fungi, including ectomycorrhizal *Scleroderma*, endophytic *Phialocephala* and *Cadophora*, and arbuscular mycorrhizal *Rhizophagus* and Glomeraceae (Figure 10; Table S7). Conversely, it significantly inhibited saprotrophic and pathogenic fungi, including *Cystobasidium*, *Coniothyrium*, *Trechispora*, and *Thelonectria* (Table S7). The increased abundance of *Scleroderma* aligns with the effects of *S. bovista* inoculation, confirming its successful establishment in the hazelnut rhizosphere. *S. bovista*, a medicinal and edible mushroom, contains ergosterol, a fungal sterol with significant antibacterial properties [52,53]. We speculate that the antibacterial compounds produced by *S. bovista* may be responsible for the observed reduction in saprotrophic and pathogenic fungi following inoculation.

## 5. Conclusions

*S. bovista* inoculation significantly enhanced root morphological parameters including root number, root tip density, total root length, root surface area, and root forks in all tested soil types. At the fungal class level, potted hazelnut seedlings exhibited increased relative abundance of *Agaricomycetes* concurrently with decreased *Sordariomycetes* abundance. Notably, *S. bovista* (*Scleroderma bovista*, *Agaricomycetes*) inoculation further amplified the Agaricomycetes predominance. Pot cultivation significantly reduced alpha diversity indices (ACE and Chao1), whereas both potting and *S. bovista* inoculation collectively diminished the Shannon diversity index. Consistent with our hypothesis, *S. bovista* inoculation, when combined with hazel root exudates, exhibited a synergistic effect: although its standalone influence on microbial community structure was less pronounced compared to potting practices, the dual action of inoculation and exudates significantly enriched symbiotic fungi (i.e., *Agaricomycetes*) while suppressing saprophytic and pathotrophic guilds (i.e., *Sordariomycetes*), thereby aligning with the proposed root growth promotion mechanism. This finding implies that root exudates synergize with *S. bovista* to facilitate probiotic recruitment and establish beneficial microbial consortia, thereby creating an ecological niche unfavorable for decomposer and pathogenic fungi. Our findings demonstrate *S. bovista*’s potential as a candidate for hazel-specific microbial inoculant development, providing both theoretical foundations for orchard management optimization and practical implications for harnessing *S. bovista* in ectomycorrhizal cultivation systems. However, it should be noted that the microbial diversity results in hazel rhizosphere soil were derived from pot experiments using two-year-old seedlings. The observed dynamics may differ from those of perennial hazelnut trees under field conditions, where complex environmental interactions and mature root systems could modulate microbial recruitment. Therefore, further validation through large-scale field trials is essential to confirm the ecological applicability of *S. bovista* inoculants in commercial orchards.

## Data Availability

The raw sequences of microbial diversity in hazel rhizosphere soil can be found in the National Center for Biotechnology Information under the accession number PRJNA1224535. The original contributions presented in this study are included in the article/Supplementary Materials; further inquiries can be directed to the corresponding authors.

## Supplementary Materials

The following supporting information can be downloaded at: https://www.mdpi.com/article/10.3390/microorganisms13051063/s1, Table S1: Physiochemical properties of soils used in the pot culture experiments; Table S2: Feature number of each sample; Table S3: Summary of raw data; Table S4: Statistics of reads count in each taxomonic level; Table S5: Statistics of taxonomic annotation; Table S6: Fungal phenotype prediction of Group 0 vs. Group 1; Table S7: Fungal phenotype prediction of Group 1 vs. Group 2.
