# Supplementary material for: Synergistic Recruitment of Symbiotic Fungi by Potting and Scleroderma bovista Inoculation Suppresses Pathogens in Hazel Rhizosphere Microbiomes
Source: Microorganisms. 2025 May 2;13(5):1063. doi: 10.3390/microorganisms13051063 (PMC12114540; doi:10.3390/microorganisms13051063)
Supplement: Supplementary file 1 [file microorganisms-13-01063-s001.zip › microorganisms-3613163-supplementary/Supplementary File/Table S1 Physiochemical properties of soils used in the pot culture experiments.pdf]

Supplementary Table 1 Physiochemical properties of soils used in the pot culture experiments

Location	Geographical coordinates	Abbreviation	pH	Organic matter (g/kg)	Total N (g/Kg)	Total P (g/Kg)	NH_4OAc-K(mg/Kg)
Yitong	E125.08, N43.56	A	7.87	21.6	124	25.9	130
Shulan	E127.01, N44.42	B	8.21	34.6	183	29.0	178
Jiaohe	E127.36, N43.72	C	7.68	26.9	245	30.0	196
Panshi	E126.17	D	6.6	40.0	461	29.1	281
Linjiang	N42.89	E	6.4	14.2	95	21.3	186
